# Preclinical good laboratory practice-compliant safety study to evaluate biodistribution and tumorigenicity of a cartilage advanced therapy medicinal product (ATMP)

**DOI:** 10.1186/s12967-015-0517-x

**Published:** 2015-05-20

**Authors:** Matthias Zscharnack, Christoph Krause, Gabriela Aust, Christian Thümmler, Frank Peinemann, Thomas Keller, Jeske J. Smink, Heidrun Holland, Jeremy S. Somerson, Jens Knauer, Ronny M. Schulz, Jörg Lehmann

**Affiliations:** Translational Centre for Regenerative Medicine (TRM), University of Leipzig, Leipzig, Germany; Fraunhofer Institute for Cell Therapy and Immunology, Leipzig, Germany; Research Laboratories, Department of Surgery, University of Leipzig, Leipzig, Germany; Centre for Biotechnology and Biomedicine (BBZ), University of Leipzig, Leipzig, Germany; ACOMED Statistik, Leipzig, Germany; co.don AG, Teltow, Germany; Department of Orthopaedics, University of Texas HSC San Antonio, San Antonio, USA

**Keywords:** Biodistribution, Tumorigenicity, Safety, ATMP, Cartilage repair, Chondrocytes

## Abstract

**Background:**

The clinical development of advanced therapy medicinal products (ATMPs), a new class of drugs, requires initial safety studies that deviate from standard non-clinical safety protocols. The study provides a strategy to address the safety aspects of biodistribution and tumorigenicity of ATMPs under good laboratory practice (GLP) conditions avoiding cell product manipulation. Moreover, the strategy was applied on a human ATMP for cartilage repair.

**Methods:**

The testing strategy addresses biodistribution and tumorigenicity using a multi-step analysis without any cell manipulation to exclude changes of test item characteristics. As a safeguard measurement for meeting regulatory expectations, the project design and goals were discussed continuously with the regulatory authority using a staggered scientific advice concept. Subsequently, the strategy was applied to co.don chondrosphere® (huChon spheroid), a tissue-engineered matrix-free ATMP of human normal chondrocytes. In both the biodistribution and tumorigenicity studies, huChon spheroids were implanted subcutaneously into 40 immunodeficient mice. Biodistribution was studied 1 month after implantation. A skin disc containing the huChon spheroid, two surrounding skin rings and selected organs were analyzed by validated, gender-specific, highly-sensitive triplex qPCR and by immunohistochemistry (IHC).

**Results:**

No human DNA was detected in distant skin rings and analyzed organs. IHC revealed no direct or indirect indications of cell migration. Tumorigenicity was assessed 6 months after huChon spheroid implantation by palpation, macroscopic inspection, histology and IHC. No mice from the huChon spheroid group developed a tumor at the implantation site. In two mice, benign tumors were detected that were negative for HLA-ABC, suggesting that they were of spontaneous murine origin.

**Conclusions:**

In summary, the presented strategy using a multi-step analysis was confirmed to be suitable for safety studies of ATMPs.

**Electronic supplementary material:**

The online version of this article (doi:10.1186/s12967-015-0517-x) contains supplementary material, which is available to authorized users.

## Background

Novel therapies using cell-based ATMPs require special safety testing strategies. These cell-based ATMPs can be classified as somatic cell therapy medicinal products (sCTMPs), tissue engineered products (TEPs), or combined ATMPs. Product development and testing must be in compliance with regulatory requirements, and the compiled data need to be reviewed by regulatory authorities such as the European Medicines Agency (EMA) in the European Union (EU) or the Food and Drug Administration (FDA) in the United States of America. For non-clinical development, conventional efficacy and safety testing strategies as established for small molecules or biopharmaceuticals have been shown to be inapplicable and unsuitable for ATMPs. Nevertheless, ATMPs must fulfill other strict testing requirements including the acquisition of safety data for authorization by regulatory authorities for clinical trial or marketing approval [1,2]. In the EU, these requirements are laid down by the European Commission in Directive 2009/120/EC, amending the overarching medicinal product Directive 2001/83/EC by implementing specific demands for ATMPs. This includes unintended biodistribution of the implanted cells, referring to their distribution or migratory potential, as well as their tumorigenic risk, referring to their potential to transform and generate tumors, and their potential immunotoxicity or immunogenicity in the patient [3].

In this work, a strategy was developed and applied to assess the biodistribution and tumorigenicity of a TEP for cartilage repair, human chondrocyte spheroids (co.don chondrosphere®) referred to here as huChon spheroids. These spheroids are a solid cell complex with a self-synthesized extracellular matrix that is formed by self-aggregation in appropriate culture conditions, and the spheroids are used for the treatment of focal lesions in articular knee cartilage [4,5]. Studies of efficacy and proof of concept including aspects of basic safety of the huChon spheroids were done under GLP conditions in an orthotopic sheep animal model to mimic the human situation [6]. In the present comprehensive two-stage safety study, an immunodeficient inbred mouse strain was chosen to evaluate subcutaneously transplanted huChon spheroids for undesirable biodistribution into circumjacent mouse tissues and selected organs as well as for signs of tumorigenicity.

The whole study, consisting of biodistribution and tumorigenicity components, was performed under GLP conditions that require detailed standard operating procedures (SOPs) for each step. Manipulation of the test item, *e.g.* by labeling with fluorescent dyes, quantum dots, ferromagnetic particles or by introduction of reporter genes such as luciferase, was regarded to be unsuitable because this may result in altered functional and safety properties of the test item [7–10]. The test item must reflect the essential features of the final ATMP produced under good manufacturing practice (GMP) or should ideally even be identical to this. Thus, methods requiring *in-vivo* labeling of huChon were excluded. In order to achieve regulatory feedback prior to initiating a time- and cost-intensive GLP-study, the project concept included several scientific advice meetings with the Paul-Ehrlich-Institute (PEI), the responsible regulatory authority for ATMPs in Germany. The GLP study design was prospective, randomized, actively controlled and performed under blinded label for the observer analyses using test items derived from the GMP manufacturing unit.

## Methods

### Study plan

#### Regulatory affairs

Equipment, materials, and methods including mouse breeding, the animal model, all assays and validation of each step were used or performed according to detailed institutional SOPs in accordance with the principles of GLP as described under § 19a, German Chemical Law and in the GLP handbooks of the WHO and OECD [11,12]. All study-relevant SOPs are available on request.

**Fig. 1:**
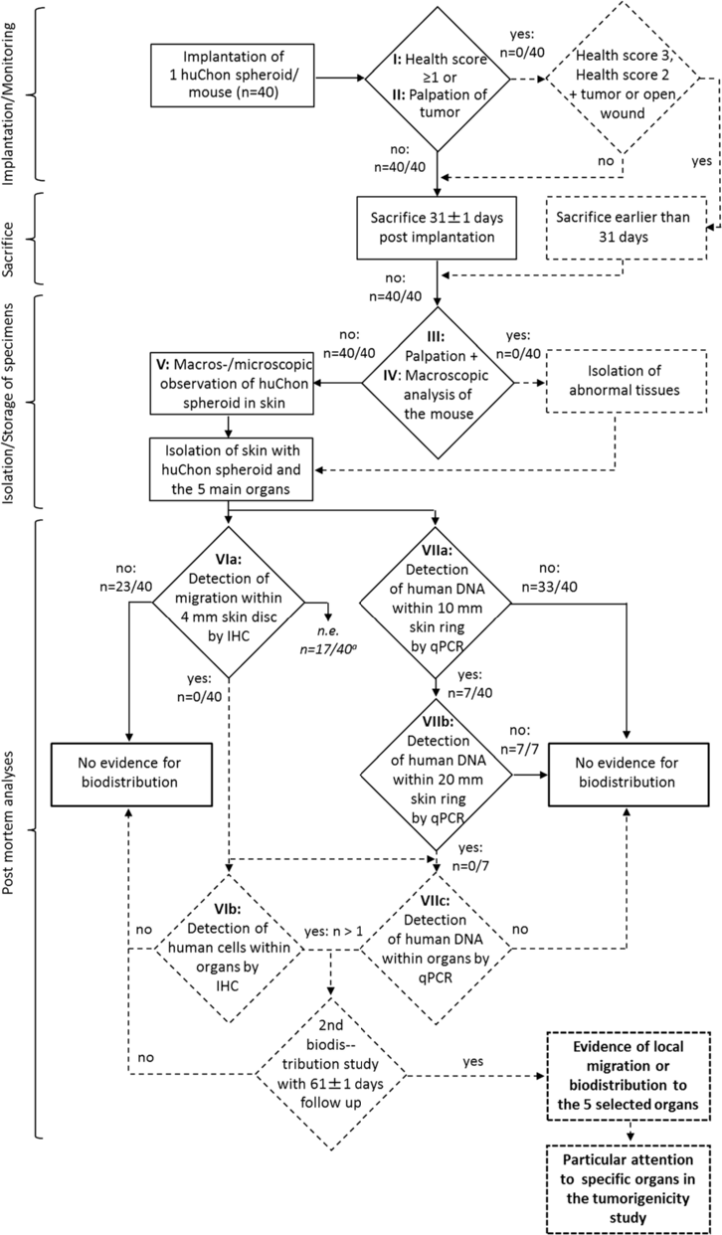
Flow chart of the biodistribution study under GLP conditions. The methods and decision points shown in continuous lines and rectangles were performed within the presented biodistribution study. Steps in dashed lines were prospectively planned but were not necessary to perform. Methods shown as a rhombus may have at least two outcomes. The Roman numerals are used in order to match each step in this flow chart with the corresponding explanation in the “Results” section. ^**a**^HuChon spheroids from five different patients were implanted, eight mice were treated per patient, each treated with 1 spheroid, and thus 40 mice were analyzed. ^**b**^In mouse MB1 (1/40) the huChon spheroid could not clearly be identified macroscopically at the implantation site. Thus, complete qPCR analyses of organs were performed. ^**c**^17 samples could not be evaluated (n.e.) by IHC due to technical reasons. The further analyses of these mice are shown in an additional flow chart in the Supplement (Additional file 3: Figure S3). In case of detection of human cells or DNA within the five selected organ/tissue types (lungs, liver, left and right kidney, spleen, and pooled local lymph nodes) the study would have to be repeated with a second cohort of 40 mice with a follow up of 61 ± 1 days. If biodistribution had been confirmed, the upstream tumorigenicity study (see below) would be performed with particular attention to the afflicted organs

**Fig. 2:**
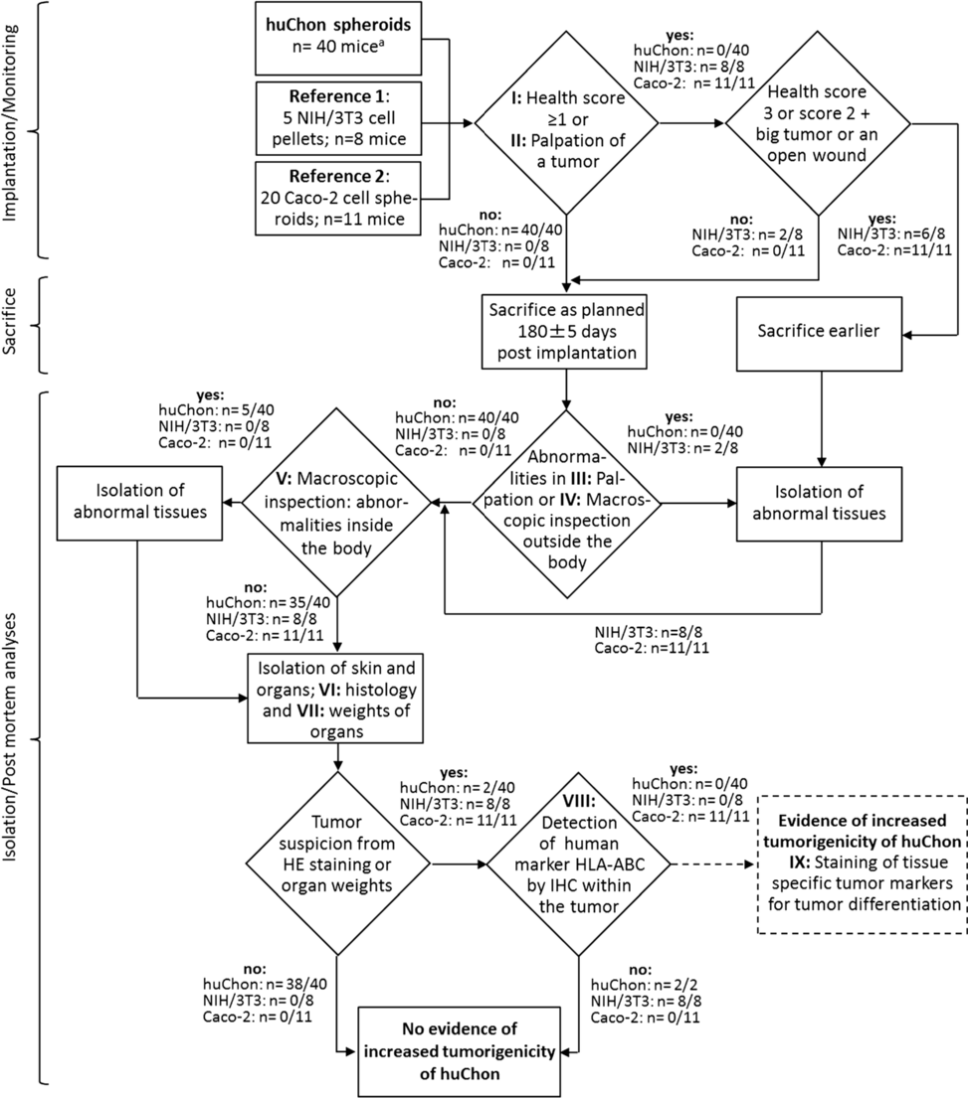
Flow chart of the tumorigenicity study under GLP conditions. The methods and decisions shown in continuous lines and rectangles were performed within the tumorigenicity study presented here. The rectangle in dashed lines refers to a potential outcome of huChon group that did not occur. Methods shown as a rhombus may have two outcomes. The Roman numerals are used in order to match each step in this flow chart with the corresponding explanation in the “Results” section. ^a^(within the topmost rectangle) huChon spheroids from five different patients were implanted, eight mice were treated per patient, 5 spheroids per mouse were implanted and thus 40 mice were analyzed. In two mice of the huChon group a benign tumor was detected. However, both tumors were anti-HLA-ABC negative suggesting that the tumors were spontaneous murine and not derived from human cells. Thus, there was no evidence for increased tumorigenicity after implantation of the huChon spheroids

The study plan was conducted in accordance with the recommendations of the Committee for Advanced Therapies (CAT), the scientific expert committee for ATMPs at the EMA [13], and the reflection paper on *in vitro* cultured chondrocyte-containing products for knee cartilage repair [6]. The study plan, documented in the flow charts (Figs. 1 and 2) and agreed upon by the responsible national regulatory authority, the PEI, was realized with the elaborated SOPs. All critical parameters of this GLP study, *i.e.* NSG mouse model, subcutaneous *vs.* orthotopic application route, dose, number of animals and patients, duration of follow-up in the biodistribution and tumorigenicity studies and general methodology of recovery of human cells were defined after discussion with the authority. This study was performed at the Fraunhofer Institute for Cell Therapy and Immunology (IZI) Leipzig as a legal GLP test facility under the study code *Fh-IZI-04-huChon (COD16/TS09)*. Any deviation from the SOPs was documented in a deviation report and approved by the study director and the head of the test facility management.

### Mouse model

All mice procedures were approved by the local ethics committee for experimental animal studies (Landesdirektion Sachsen, A06/11) and thus were in accordance with the NIH Guide for the Care and Use of Laboratory Animals. NOD.Cg-*Prkdc*^*scid*^*Il2rg*^*tm1Wjl*^/SzJ (NSG) mice (Charles River, Margate, UK) are recommended for examining biodistribution of human xenografts [14]. Female NSG mice, 7–9 weeks of age, were housed under special pathogen-free conditions in individually ventilated cages and received food and water *ad libitum*.

### Test and reference items

**Fig. 3:**
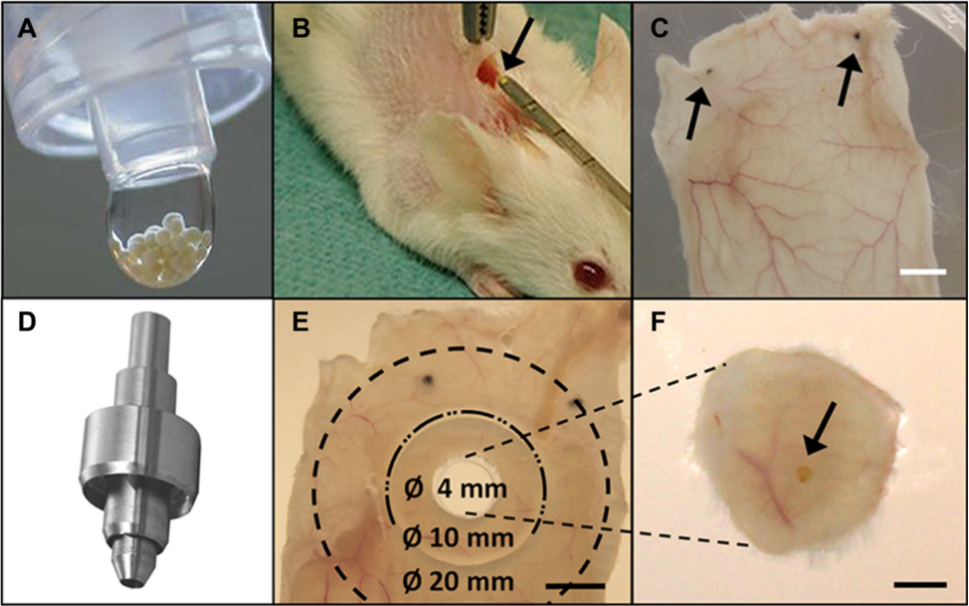
huChon spheroids, their implantation and the isolation of skin areas in the biodistribution study using a three-part punch. HuChon spheroids in a drop of a syringe ready for implantation after GMP manufacturing **(a)**. Subcutaneous implantation of a single huChon spheroid (arrow) by a micro-spatula into the back of the NSG mouse, 1 cm caudally from the occipital pole **(b)**. The spatula had marks every 0.5 cm to ensure correct implantation. The isolated back skin with the two tattoo points from the former wound edges as orientation were spreaded for punching in a Petri dish **(c)**. Three-part punch, developed for this study **(d)**. Closer look with the labeling of the three skin areas, the inner 4-mm disc has been punched out **(e)**. Representative example of such a punched out 4-mm disc with the spheroid (**f**, arrow). Scale bars: **c**: 10 mm, **e**: 4 mm, **f**: 1 mm

Chondrocyte 3D spheroids (Fig. 3a) produced *in vitro* from human articular chondrocytes (*co.don chondrosphere*®, co.don® AG, Teltow, Germany), referred to here as huChon spheroid, were examined as an ATMP. Chondrocytes were taken exclusively from female patients who underwent the initial surgery for the autologous chondrocyte transplantation procedure. The cells were cultivated as monolayers for 21–33 days. The spheroids with an average diameter of 500 ± 70 μm were manufactured by co.don^®^ as described previously [15]. Briefly, huChon spheroids were prepared with 2 × 10^5^ chondrocytes from passage 2 or 3 and cultured for an additional 21–31 days. The production-relevant quality and safety tests were performed under the supervision and on the premises of co.don similar to the procedure required for GMP. The huChon spheroids complying with the requirements of the quality control program were transferred to the GLP test facility. The mouse embryonic fibroblast cell line NIH/3T3 and the human colorectal adenocarcinoma cell line Caco-2 (both ATCC; LGC Standards GmbH, Wesel, Germany) were used for references of the tumorigenicity study. Aggregation of NIH/3T3 was accomplished by centrifugation of 0.2 × 10^6^ cells at 780 × g for 5 min and by cultivation of these pellets for 24 h. Spheroid analogues from Caco-2 cells were produced according to the co.don chondrosphere^®^ protocol.

### Implantation of spheroids

The spheroids or spheroid analogues were implanted subcutaneously into the back of the mice 1 cm caudally from the occipital pole under anesthesia with 65 mg/kg ketamine (Bela-Pharm, Vechta, Germany) and 13 mg/kg xylazine (Bayer HealthCare, Leverkusen, Germany). A 0.5 cm skin incision was made and the spheroids were inserted by means of a micro-spatula (Fig. 3b). The wound edges were tattooed with a sterile 26-gauge needle and black tattoo paste (Peter Hennes, Haan, Germany) for locating the implantation site.

### Biodistribution

The complete process of the prospective biodistribution study, including multi-step analysis and important decision points, is illustrated in a detailed flow chart (Fig. 1). HuChon spheroids of five female patients were analyzed. One huChon spheroid was implanted per mouse. Eight mice were treated per patient, thus 40 mice were analyzed. After implantation of huChon spheroids, the mice were inspected daily, weighed and palpated weekly, and their health was scored using a system modified according to te Velde *et al.* [16], and the German Society of Laboratory Animals (score 0: normal; score 1: shaggy fur; score 2: withdrawn behavior, eating less, humped-up posture; score 3: apathetic behavior, weight loss of > 20 %). The break-off criteria were: health score 3 or health score 2 combined with a tumor or an open wound. After sacrifice, which was performed 31 ± 1 days after implantation, the area surrounding the implantation site was judged by macroscopic inspection and the whole mouse body was palpated. Subsequently, a 3.5 × 5 cm skin area was explanted from the back, spread on a petri dish (Fig. 3c) and the huChon spheroid was observed macroscopically and microscopically using an inverse microscope (Axiovert, Zeiss, Oberkochem, Germany). Using a three-part biopsy punch developed in house (Fig. 3d), a 4-mm diameter skin disc and two circumjacent ring punches, 10-20 mm diameter (Fig. 3e), were obtained. The 4-mm disc (Fig. 3f), with the spheroid in its center and frozen at −80 ± 5 °C in cryomolds with Tissue-Tek® (Jung, Nussloch, Germany), was used for IHC of potentially migrated human cells. The circumjacent 10-mm ring within a radius of 2-5 mm from the implant was examined for the presence of human DNA by qPCR. The circumjacent 20-mm ring within the radius of 5-10 mm distance from the implant was cryopreserved in cryotubes at −80 ± 5 °C for optional qPCR analysis if the 10-mm ring was tested positive for human DNA. Further, five different organs—lungs, liver, the two kidneys, spleen, and local lymph nodes (pooled from each mouse)—were isolated, weighed and one third of each tissue was frozen in Tissue-Tek® at −80 ± 5 °C for optional IHC. The remaining organ portions were frozen at −20 ± 5 °C for optional qPCR.

In case of evidence of local migration or detection of human cells/DNA within the analyzed organ types, the study would have to be repeated with a second cohort of 40 mice, with a follow up of 61 ± 1 days. If biodistribution had been confirmed, an upstream tumorigenicity study would be performed with particular attention to the affected tissues or organs.

### Tumorigenicity

The complete process of the prospective study is illustrated in a detailed flow chart (Fig. 2). HuChon spheroids of five additional female patients were analyzed. Five huChon spheroids were implanted per mouse. Eight mice were treated per patient, thus 40 mice were analyzed. For reference group 1, 20 Caco-2 spheroids, each containing 0.5 × 10^6^ cells, were implanted per mouse (n = 11) to ensure tumor formation [17]. As reference group 2, prospectively planned as a negative reference, five spheroid analogues with a total of 1 × 10^6^ NIH/3T3 cells were implanted per mouse (n = 8). The planned husbandry period was 180 ± 5 days. After sacrifice, the mice were palpated and macroscopically inspected for tumors. Tumor size was measured with a caliper to calculate volume. Mice were dissected and any tumors or abnormalities at the implantation site or organs were prepared and frozen in cryomolds with Tissue-Tek® at −80 ± 5 °C. The selected organs were isolated, weighed and frozen as described for biodistribution. Serial sections of the tumors and suspicious areas were analyzed by HE and HLA-ABC staining.

### DNA extraction

To prevent any contamination with human female cells or DNA, all experiments were performed by male staff only. The complete 10-mm skin ring and the samples of isolated organs were weighed, mechanically minced with scalpels and lysed with 0.5 μg proteinase K (Promega, Mannheim, Germany) per mg tissue under continuous shaking at 56 °C for 90 min. The DNA was automatically extracted using the Maxwell® 16 Mouse Tail DNA Purification Kit at the Maxwell 16 IVD Instrument (both Promega) in the “standard elution volume” mode. First, the extraction process was validated. Intra-assay variation, *i.e.* the coefficient of variation (CV) between three different DNA extractions from a single skin lysate in the same run, was determined in triplicate. The procedure was repeated on three different days. The overall intra-assay variability was 2.85 ± 0.8 %. Inter-assay variation was examined between the same three runs. The CV value was 0.94 ± 2.1 % (Table 1).

**Table 1:** Validation of the DNA extraction process

A)	DNA concentrations and the resulting intra-assay variation between three different DNA extractions from a single skin lysate in the same run. “Eluate 1–3” are different extractions using three different Maxwell cartridges within one run. The values were determined in triplicate (assay 1–3 at three different days)					
	DNA concentration [ng/μl]					Intra-assay variability [%]
	Eluate 1	Eluate 2	Eluate 3	Mean	SD	
Assay 1	219.0	231.6	223.8	224.8	6.36	2.83
Assay 2	219.7	234.7	219.8	224.7	8.63	3.84
Assay 3	219.9	225.8	217.7	221.1	4.19	1.89
Mean						2.85
SD						0.8
B)	Mean DNA concentrations of the validation runs at three different days (assay 1–3) and resulting inter-assay variation.					
	DNA concentration [ng/μl]					Inter-assay variability [%]
	Assay 1	Assay 2	Assay 3	Mean	SD	
	224.8	224.7	221.1	223.5	2.10	0.94

To test for possible saturation of the DNA extraction when increasing the amount of skin lysate per cartridge, DNA was prepared from 5 mg up to 69 mg skin in 3 mg steps. Within this range, the amount of extracted DNA per cartridge increased linearly. Thus, per cartridge up to 50 mg tissue sample were used.

If two or more cartridges were used for a punched skin ring or tissue sample the extracted DNA was pooled. The volume of each DNA was determined for calculation of possible number of PCR reactions.

### Triplex qPCR

Triplex qPCR was performed using the Plexor^®^ HY Assay (Promega), which offers a combination of simultaneous quantification of human autosomal and Y-chromosomal DNA, with a LightCycler^®^ 480 instrument (Roche Diagnostics, Mannheim, Germany). Multi-copy sequences on chromosome 17 and on the Y chromosome were targeted in order to detect human autosomal DNA or male DNA, respectively. Because implantation of the huChon spheroids from female donors and the downstream qPCR analysis were exclusively done by male lab personnel, the gender-specific primers allowed discrimination of donor DNA from DNA contaminated by lab personnel. A third novel DNA sequence was included in the primer mix as an internal PCR control (IPC). In each run, a negative control without template and a calibration curve consisting of seven log_5_ dilution steps created from the Plexor^®^ HY male gDNA standard calibrator were included in triplicate.

Each PCR reaction contained 10 μl Plexor® HY 2x master mix, 1 μl Plexor® HY 20X primer/IPC mix and 9 μl sample DNA template. From each skin ring or tissue sample, at least 1/3 of the total extracted DNA volume was used for qPCR. That means 1/3 of DNA volume divided by 9 determined the minimal number of PCR reactions per skin ring or tissue sample. The minimal number of qPCR reactions per skin sample was 19. The maximum number was 102 reactions. Data were analyzed using the Plexor® analysis software (Promega). Assignment of reference standard reactions for the calibration curve or negative control and adjustment of expected target melt temperatures were done according to the Plexor® HY protocol. Three channels were estimated for each qPCR reaction: “IPC”, “human autosomal” and “male”. “IPC” must be positive, otherwise the analysis for this reaction was stopped. A reaction was considered positive for the “human autosomal” and/or “male” channel if extrapolated by the Plexor® analysis software.

### Determination of detection limits in DNA extraction combined with qPCR

In order to evaluate the sensitivity and specificity for human female DNA, decreasing numbers of human chondrocytes were transferred into tubes containing up to 50 mg fresh mechanically-minced sterile murine skin to undergo further proteinase K lysis. DNA was extracted and used completely for qPCR. Reference standard reactions were performed in parallel. The analysis was carried out in triplicate on three consecutive days. At the end, the percentage of positive qPCR results of one murine skin sample substituted with a decreasing number of human female chondrocytes was calculated for the channels “human autosomal” and “male” to determine sensitivity and specificity, *i.e.* the contamination rate. Reactions positive for both channels were considered to have been contaminated by the male staff and were not included in calculations.

**Fig. 4:**
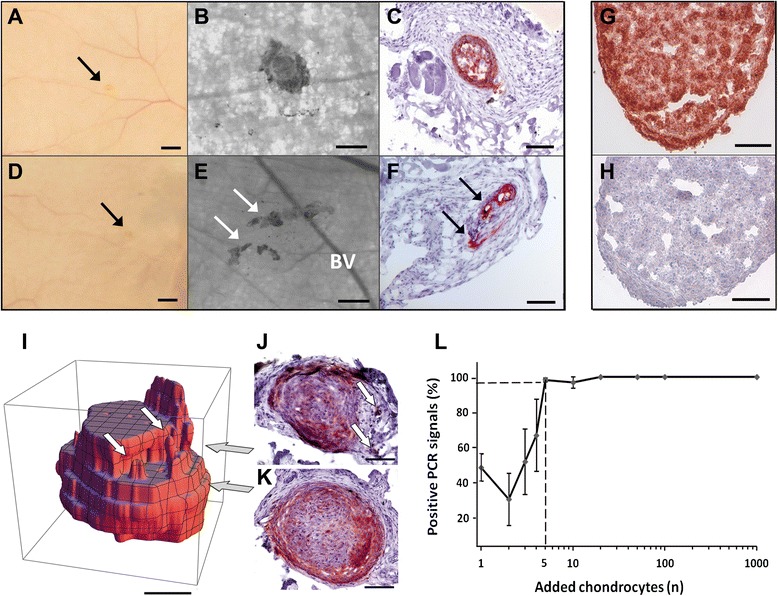
Biodistribution study: Detection of the huChon spheroids within the mouse skin. Detection of implanted huChon spheroids within mouse subcutis; the figures show a representative example of a normally shaped huChon spheroid (**a–c**) and a fragmented spheroid (**d–f**). The huChon spheroid was macroscopically visible as faint yellowish stain (**a**, arrow). Light microscopic view of the spheroid **(b)**. HLA-ABC staining of this specimen (**c**, red-brown staining). Haematoxylin was used as a counterstain. The fragmented huChon spheroid is only very weakly visible macroscopically (**d**, arrow). A Light microscopic view of a fragmented spheroid is shown in **(e)**. BV: Blood vessel. HLA-ABC staining of a fragmented spheroid is shown in **(f)**. The arrows indicate the two fragments. As positive control, HLA-ABC staining of a single huChon spheroid without mouse tissue was used **(g)**. IgG2a isotype control of this specimen is shown in (**H**). Scale bars indicate 100 μm (**g**, **h**), 200 μm (**b, c, e**, and **f**) or 500 μm (**a, d**). A 3D reconstruction of a representative huChon spheroid from nine serial sections was performed **(i)**. The digitized images were registered onto another to realign adjacent slices. Afterwards, each image was binarized to a separate background. An anisotropic Gaussian filter was used to smooth the data for visualization and afterwards the surface of the volume was reconstructed using the ListContourPlot3D algorithm implemented in Mathematica 9.0.1. Depending on the section level (grey arrows), this huChon spheroid appeared fragmented **(j)** or not **(k)**. The white arrows in **(i)** and **(j)** indicate “bulges” of the spheroid. Scale bars indicate 100 μm. The determination of the lower detection limit for DNA extraction and the Plexor® HY PCR (**l**). The sensitivity and reproducibility of the DNA extraction and qPCR were tested in parallel by adding increasing numbers of human chondrocytes to 50 mg mouse skin tissue. Five cells represented the lowest cell number at which almost all of the reactions were reproducibly positive

In a first series, 1, 10, 100 or 1000 cells were added (n = 60 qPCRs/cell number). All qPCR analyses were positive using 100 or more cells. In a second series 5, 10, 20, 50 or 100 cells (n = 60 qPCRs/cell number) were added. All qPCR analyses were 100 % positive using 20 or more cells. In a third series 1, 2, 3, 4, 5 or 10 cells (n = 120 qPCRs/cell number) were added. Human autosomal DNA was detected in 98.3 ± 1.2 % with five cells (Fig. 4l). For less than five cells, detection was not reliable and reproducible. Thus, 5 cells at minimum were required within one DNA extraction and qPCR process to detect human autosomal DNA reproducibly.

Furthermore, the percentages of positive qPCR results for one channel were calculated. The threshold percentage for a DNA sample to be considered positive for “human autosomal” was calculated as [100−2x ratio of (number of negative reactions/number of all reactions)], therefore adjusting the threshold downwards (unadjusted values). Because the number of negative reactions was two within 120 reactions, the percentage of positive qPCR results was 96.6 %. That means a mouse tissue was considered positive for at least five human cells when 96.6 % of the reactions were positive for “human autosomal”.

The threshold percentage for a DNA to be contaminated overly with male DNA to be undoubtedly identified as female was calculated as [2x ratio (number of positive reactions in channel “male”/all reactions)]. It was 30 %, as there were 18 positive reactions in channel “male” within 120 reactions. If less than 30 % of the reactions of a sample were also negative for the “male” qPCR channel, female DNA was present. Male DNA was detected in 3.1 ± 1.2 % of all validation qPCR analyses, whereby 93.5 ± 5.1 % of these reactions correlated with human DNA and can thus be explained by a contamination of male staff. That means 6.5 ± 5.1 % of the qPCR analyses positive for male DNA were false positive.

### qPCR statistics

For each murine tissue sample, we determined whether it contained a certain number of human cells. PCR statistics involved the application of a two-sided confidence interval to the calculated percentages of positive qPCRs results per DNA sample and channel, extending the calculated value both upwards and downwards by a range of uncertainty in which the value might also be found. Because in murine male tissue samples human female chondrocyte DNA should be detected which was extracted by male staff, the criteria for evaluating the channels “human autosomal” and “male” were made stricter by adjusting the channel “human autosomal” values upwards and the channel “male” values downwards for the uncertainty. These adjusted percentages were compared with channel-dependent threshold percentages resulting from validation experiments. Reaching or exceeding the channel “human autosomal” threshold allowed this DNA sample to be judged positive for a minimum of human cells during DNA extraction which was determined during qPCR validation. If the channel “human autosomal” was positive but simultaneously the threshold of the “male” channel was not reached (undershooting), the cells were additionally categorized as female. If the channel “male” threshold was exceeded, the DNA was considered to be overly contaminated with male DNA derived from lab personnel.

### HE staining and immunohistochemistry

For HE staining, the 10 μm cryosections were fixed with 10 % paraformaldehyde for 15 min, washed twice with deionized water and stained with filtered hematoxylin solution (Morphisto, Frankfurt, Germany) for 10 min. After washing with tap water for 15 min and a short washing step with distilled water, the sections were stained with 1 % eosin (Morphisto) for 3 min.

In order to detect potentially migrated human cells within the 4-mm skin disc with high sensitivity and specificity by immunohistochemistry, the staining protocol was optimized. The particular challenge was to achieve specific staining of the spheroids, fragments or single chondrocytes without any background staining of murine tissue. The epidermis often shows non-specific staining due to its expression of endogenous peroxidase [18]. Several blocking steps including inhibition of the endogenous peroxidase, incubation of the tissue sections with human serum before incubation with the primary anti-human antibody, and adding species-specific serum to the secondary antibody resulted in distinguishable staining in pilot batches (Additional file 1: Figure S1).

At the end, the following optimized staining protocol was applied under GLP conditions: The complete 4-mm disc was cut vertical to the epidermis into serial 10 μm cryosections. To reduce the sample size, only every second section was labeled with a serial number and immunohistochemically stained. Prior to the staining, the sections were fixed with acetone for 10 min, incubated with 0.3 % H_2_O_2_ in PBS for 30 min to block endogenous peroxidase activity, washed twice with PBS and incubated with 1 % human AB serum (Sigma-Aldrich, Deisenhofen, Germany) for 15 min. The serum was removed and the sections were incubated with the primary anti-HLA-ABC mouse monoclonal antibody (clone W6/32) or alternatively with an IgG2a isotype control antibody (both DakoCytomation, Hamburg, Germany) for 90 min at room temperature. Then, the sections were washed three times and incubated with horseradish peroxidase-conjugated goat anti-mouse IgG (Dianova, Hamburg Germany), supplemented with 1.5 % goat serum, for 45 min at room temperature. After three washing steps the sections were developed with 3–amino–9–ethyl–carbazol (AEC) as substrate (Sigma-Aldrich). The nuclei were counterstained with Mayer’s hematoxylin (DakoCytomation). Spheroid cryoslides of the relevant patients were used in parallel as positive controls.

The slices were assessed blindly by two independent experts. The primary aim was to reveal direct signs of active migration such as a corona-forming spread of chondrocytes starting from the implanted spheroid. The secondary aim was to find indirect signs of migration resulting from migrated cells. This included the occurrence of tumors, infiltration of neutrophilic granulocytes, and induction of necrosis or apoptosis seen as cell shrinkage, formation of apoptotic bodies or pyknotic chromatin [19].

After optimization and validation of immunohistochemistry, a GLP-compliant SOP was generated. Reproducibility of the method was tested by validation stainings carried out on three consecutive days.

### Karyotyping

Karyotyping was not performed under GLP conditions. Chromosomes were prepared from NIH/3T3 cells using standard cytogenetic techniques [20]. For karyotyping, we analyzed metaphases of cultured cells using GTG-banding [21] and the SKY-technique according to the manufacturer’s instructions at chromosome spreads. Twenty-five metaphases (12 GTG- banding, 13 SKY) were analyzed two times: within the first and within the 14th passage (at the time of implantation). Additionally, small pieces of NIH/3T3-derived mouse tumors were transferred to culture flasks. The resulting outgrown cells were karyotyped as well.

### Statistics

Results for all variables are presented as mean ± standard deviation (SD). Patient group differences (8 mice per patient group) in the mouse body and organ weights as well as their changes versus baseline were investigated by analysis of variance (ANOVA). Two-group comparisons of distributions were performed by *t*-test. For all analyses, *p*-values <0.05 were regarded as significant. With exception of Tukey’s post-hoc test, which addresses multiplicity of all pairwise comparisons between subjects in ANOVA, no correction in terms of multiple testing was applied.

Regarding the evaluation of 40 animal organs by qPCR out of 102 organs (corresponding to 7 out of 17 mice, see Additional file 1: Figure S1) we followed ISO 2859-I, Tables I and II. Regarding the evaluation of 40 animal organs by qPCR out of 102 organs (corresponding to 7 out of 17 mice, see Additional file 1: Figure S1) we followed ISO 2859-I, Tables I and II. The operational characteristic used in ISO 2859-I could also be estimated by applying a formula for binomial distribution calculating probabilities 1-α (typically =95 %) or β (typically 20 (10 %) in dependence on underlying probability of a positive event p, observed events (=0) and sample size N e. g. by using MS Excel (Formula BINOM.VERT(0 [=observed events]; N [=sample size]; p [=underlying probability]; 0 [cumulative = no]).

## Results

### Test strategy

The complete established two-staged test strategy is summarized in two flow charts (Figs. 1 and 2), which provide an overview for the whole, multi-step process with important decision points. The application of several analysis methods (Fig. 1, methods I–VII for biodistribution study and Fig. 2, methods I–IX for tumorigenicity) ensured a high degree of significance and level of security for the final evaluation of the results. Further, the use of a multi-level procedure, in which more in-depth analyses are triggered by a positive result in the previous analysis, allows for cost efficiency.

### Biodistribution study

The flow chart in Fig. 1 summarizes both the prospective planned biodistribution study as well as the obtained results.

### Normal health state and weight progression with no abnormalities

After implantation of the huChon spheroids, the mice did not show any signs of inflammatory reactions. All wounds caused by the implantation procedure healed well. From the day of implantation to sacrifice after 31 ± 1 days, body weights increased in all animals. The mean body weight of all mice was 21.9 ± 1.1 g at the day of spheroid implantation and 24.7 ± 1.2 g at the day of sacrifice. None of the animals showed weight loss of more than 20 %, health score > 0, or abnormalities with routine palpation during husbandry (Fig. 1; method I, II). Thus, all 40 mice were sacrificed 31 ± 1 days after implantation and not earlier. None of the mice showed any abnormality with routine palpation and macroscopic inspection on the day of sacrifice (Fig. 1; III, IV). The macroscopic appearance of organs was normal.

### ATMP was retrievable at one month post implantation but partially fragmented

After isolation and spreading of the back skin, huChon spheroids were macroscopically visible at the implantation site in 39 out of 40 (39/40) mice (Fig. 1; V). In one mouse (MB1) the exact location of the spheroid could not clearly be identified by eye. This mouse was considered as potentially positive for biodistribution. Thus, complete qPCR analyses of the 10-mm and 20-mm rings and the selected organs were performed.

Overall, the huChon spheroids were weakly visible at the implantation site and appeared macroscopically with a bright yellow color (Fig. 4a, d). Thus, they could hardly be discriminated from the surrounding mouse tissue. In contrast, inverse-microscopy allowed for better discrimination (Fig. 4b, e). In 5/39 mice the spheroids were found to be fragmented (Fig. 4e).

### No histological signs of biodistribution

To observe possible histological signs for local migration of human chondrocytes out of the huChon spheroid into the surrounding skin area *in situ*, IHC of HLA-ABC was performed to detect the presence of human cells (Fig. 1; VIa). From the 40 analyzed 4-mm discs containing the huChon spheroid, 2,556 sections were stained and assessed blindly. The positive HLA-ABC staining (Fig. 4g) and the negative IgG2a isotype-control (Fig. 4h) of huChon spheroid sections demonstrated the specificity of the staining. The huChon spheroids within the serial sections were shaped normally or fragmented (Fig. 4c, f). In both cases we did not observe any signs of active cell migration such as a cell migration path or a radial spread of human chondrocytes from the huChon spheroids. An indirect indication for human cell migration such as local inflammation was also not observed. The fragmentation of the huChon spheroid in 5/39 mice (Fig. 4e, f) was caused either by mechanical stress during subcutaneous implantation, or the HLA-positive “cell islets” outside the spheroid result from a deformed but intact spheroid as demonstrated by 3D reconstruction of serial sections (Fig. 4i–k). Because no indications of local migration were observed, detection of human cells by IHC in organs (Fig. 1, VIb) was not mandatory according the study plan.

However, as a limitation of the fixed safety testing strategy, only 23/40 of the 4-mm skin discs could be included for evaluation of biodistribution by IHC. The remaining 17 samples were excluded due to the suspicion of technical uncertainties. This included slipping of the spheroids from the inner 4-mm disc into the 10-mm ring and loss of spheroids during isolation throughout the punching of the inner 4-mm disc because of the very soft consistency of this test item and its loose association with the mouse fascia. However, all 40 mice were evaluable in the qPCR analysis, performed in parallel to the IHC analyses.

### qPCR revealed no indications for biodistribution into skin or organs

Using the validated Triplex qPCR, the 10-mm skin ring was examined first (Fig. 1, VIIa). A total of 760 qPCR reactions were performed. According to the determined detection limit of ≥ 5 cells, 33/40 areas were negative and 7/40 were positive for human DNA. Two out of these seven samples were also positive for male DNA probably through contamination with human DNA by male staff, detected by the implemented gender control. According to the biodistribution flow chart these seven samples were classified as potentially positive for local migration and subsequently the 20-mm ring was analyzed by qPCR (Fig. 1; VIIb). This included a total of 386 qPCR assays. In none of these samples human DNA was detected, suggesting there was no migration. Thus, in our opinion, the positive 10-mm rings were most likely caused by slipping of the soft spheroids or spheroid parts during punching from 4-mm disc into the adjacent area.

The qPCR analyses that were performed met the requirements established in the biodistribution flow chart. However, due to remaining uncertainties for the 17 animals, which were not evaluable by IHC (see above), we decided to perform additional qPCRs from a random sample of 7 out of these 17 animals (corresponding to 40/102 organs, see Additional file 1: Figure S1).

For the determination and evaluation of this random sample we followed the procedure as described in ISO 2859-I, Tables I and II. In brief, using an acceptance quality limit of 0.1 (error rate in % according to a 5 % risk for assuming migration even if it does not occur [=alpha error]) and normal inspection, 32 organs would need to be investigated. None of these organs would be allowed to test positive, otherwise the null hypothesis would be rejected.

Analyzing the 40 organs from these 7 mice by qPCR we found no positive result, thus the null hypothesis was acceptable. According to the operational characteristics of this statistical test design, a limiting quality value of 4 % (5.5 %, 7.2 %) was obtained corresponding to an error rate of 80 % power (90 %, 95 %) to detect migration even if it does not occur (=beta error).

In summary, there was no suspicion of biodistribution of spheroid-derived human chondrocytes using qPCR.

### Final evaluation of the biodistribution study

Overall, examining biodistribution by the applied combined methods ensured high sensitivity and specificity for testing a human ATMP in immunodeficient NSG mice.

Because there was no evidence of local migration into the adjacent skin and for biodistribution into the tested organs, the subsequent tumorigenicity study did not need to be performed with particular attention to any special tissues or organs.

### Tumorigenicity study

The flow chart in Fig. 2 summarizes both the prospectively planned tumorigenicity study as well as the obtained results.

### No mice in the huChon spheroid group developed a tumor at the implantation site

**Fig. 5:**
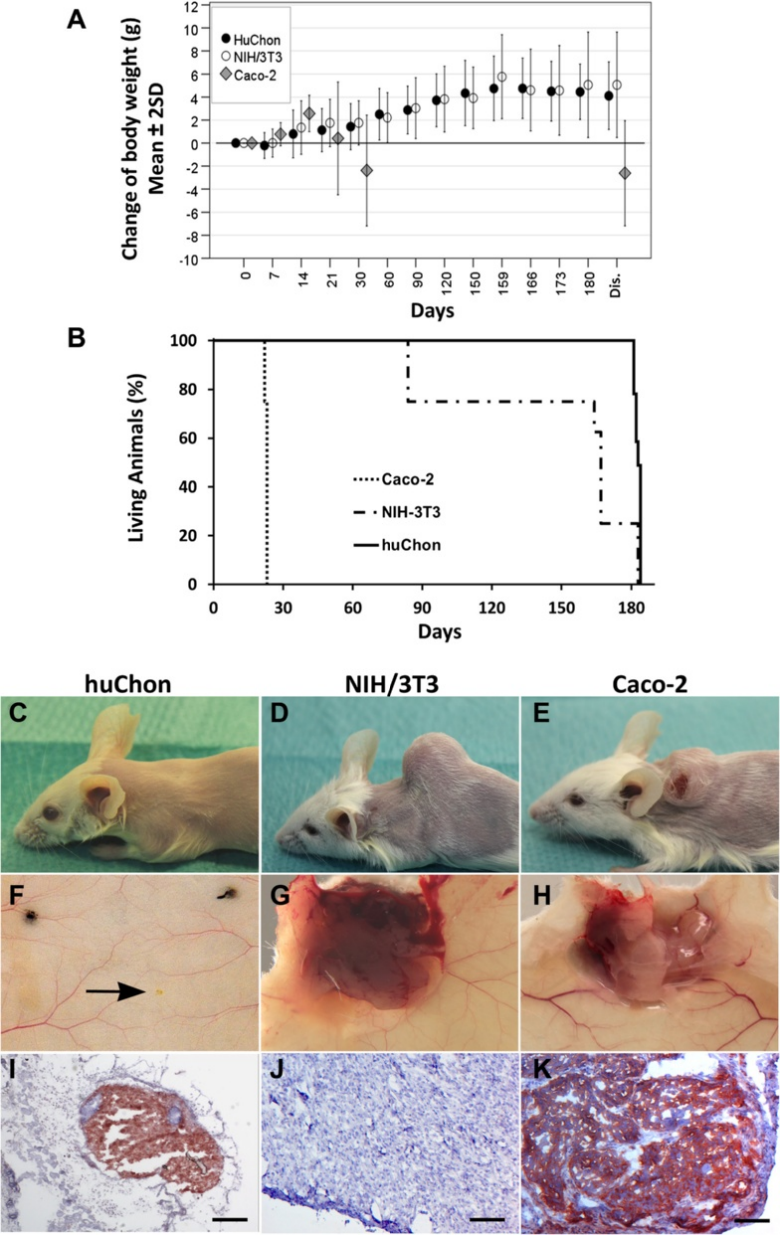
Tumorigenicity study: Comparison of the huChon spheroid group with the reference groups. Changes of mouse body weight over time normalized to the body weight at implantation of the test item **(a)**. Note: ± 2 SD in **(k)** covers approx. 95 % of all data in the sample. Dis.: Time point of dissection. Percentage of living animals of the three study groups to show the fraction of mice living for a certain amount of time after implantation **(b)**. Animals of the Caco-2 and NHI/3T3 group were sacrificed just before the planned study time according to the defined break-off criteria. Macroscopic view at the prepared implantation site of one representative mouse of each group before (**c–e**) and after (**f–h**) dissection. No tumor but the implanted huChon spheroid was located at the implantation site (**f**, arrow), whereas tumors were observed in the NIH/3T3 **(g)** and Caco-2 groups **(h)**. Representative anti-HLA-ABC staining of a section through the implantation site of each group (**i–k**). The huChon spheroids and the tumors derived from human Caco-2 but as expected not from murine NIH/3T3 cells showed a positive HLA-ABC staining. Bars indicate 200 μm

After implantation of the huChon spheroids, the mice did not show any signs of inflammatory reactions and all wounds healed well. None of the animals showed a health score > 0, weight loss (body weights increased by 6.6 ± 1.8 g over time, corresponding to 30.5 ± 8.6 % increase of the body weight (Fig. 5a) or abnormalities with routine palpation (Fig. 2; method I, II). Thus, all mice were sacrificed 180 ± 5 days post implantation. A tumor was not found by routine palpation or macroscopic inspection in any mice on the day of sacrifice (Fig. 2; III, IV). In contrast, all mice of the Caco-2 positive reference group were required to be sacrificed just 22 or 23 days post implantation (Fig. 5b) according to the defined break-off criteria. The health score of these mice was 2 or 3. All mice of this group developed a tumor at the implantation site, mostly combined with an open wound. The growing tumors exhibited a highly catabolic metabolism because mouse body weights decreased from day 14 onwards (Fig. 5a). No other tumors were detected in these mice.

Unexpectedly, all animals of the NIH/3T3 group developed tumors at the implantation site and had a health score of 1 or 2. According to the defined break-off criteria 6/8 mice had to be sacrificed between day 84 and 167 post implantation. Only two mice could be kept alive for 180 ± 5 days. After sacrifice, other tumors were not detected by palpation.

### Karyotyping of NIH/3T3 cells

Karyotyping was performed of the NIH/3T3 cell line at different cultivation times to authenticate it and to clarify why these murine cells formed tumors in NSG mice. Analyzing 25 metaphase cells by GTG-banding and SKY for each probe, we could confirm the predominant occurrence of the hypertriploid karyotype in NIH/3T3. In the first analysis of the recultivated cells after arrival, we were able to identify the novel recurrent nonreciprocal unbalanced translocations t(1;15)(C2;?) and t(7;4)(?;?). Additionally, specific structural chromosomal aberrations such as terminal deletions del(4)(B2), del(7)(F1), and del(12)(C2) were detected as single events. Most of these chromosomal aberrations were confirmed at passage 14, the time of implantation. Recurring terminal deletion del(4)(B2) was found in 57 % of the analyzed metaphases. The incidence of this deletion increased up to 100 % in explanted tumors of the NSG mouse. The terminal deletion del(7)(F1) additionally occurred in 83 % of the analyzed metaphases of the tumors. In particular, these two deletions might have contributed to tumor formation.

Thus, the accumulation of chromosomal deletions during cultivation of NIH/3T3 cells and incubation in NSG mice provides evidence for a tumorigenic geno- and phenotype of this cell line.

### Dissection revealed possible tumor-related changes in tissues of the huChon spheroid group

The implanted huChon spheroids were detected at the implantation site but no tumor formation or other abnormality was observed (Fig. 5c, f, i).

**Fig. 6:**
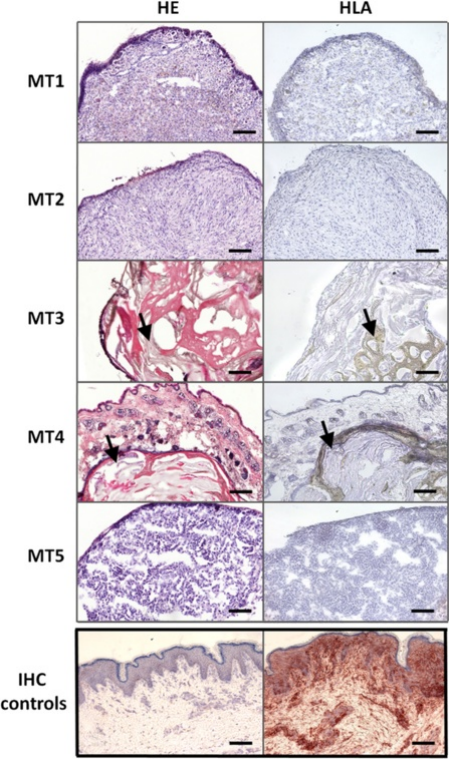
Tumorigenicity study: HE and HLA-ABC staining of serial sections of abnormal organs or tissues from five mice of the huChon group. MT1: liver, hepatoma (benign); MT2: colon, small neurinoma (benign); MT3 and MT4: abdominal skin, hair retention cysts (no tumors); MT5: pulmonary lobe, microfocal, benign malformation (no tumor). Thus, only the suspicious areas of mice MT1 and MT2 were classified histopathologically as tumors. However, these tissues were HLA-ABC negative suggesting that they are spontaneous murine rather than human tumors. IHC controls: Sections of human skin stained with the IgG2a isotype control (left) and with the anti-HLA-ABC (right) antibodies served as staining controls. Bars indicate 200 μm

During mouse dissection, organs or tissues of 5/40 mice showed possible tumor-related abnormalities (Fig. 2, step V; Fig. 6) that needed further histopathological analysis by HE and HLA-ABC staining (Fig. 2, steps VI, VIII): In the liver of mouse 1 of the tumorigenicity study (MT1), in the intestine of MT2, in the abdominal skin of MT3 and MT4 and in the right pulmonary lobe of MT5. Two of five of these tumor-suspicious organs or tissue sites were subsequently classified by histopathology as tumors. In the liver of MT1, the suspicious area showed histological signs of a hepatoma with small necroses whereas the suspicious area in the colon of MT2 was classified as a small neurinoma, both benign tumors. However, both tumors were HLA-ABC negative (Fig. 6) suggesting that they were spontaneous murine rather than human chondrocyte-derived tumors. The suspicious areas in the skin of animals MT3 and MT4 turned out to be hair retention cysts, not tumors. The observed abnormality in the lung of MT5 was assessed as a microfocal, benign malformation. However, all five conspicuous mice got huChon spheroids from five different donors. Therefore, a link between donors and tumorigenesis did not exist.

In the Caco-2 and NIH/3T3 groups, apart from the tumors at the implantation site (Fig. 5d, e, g, and h), no other tumors were observed during mouse dissection. The average volume of the tumor at time of sacrifice was 1.25 ± 0.56 cm^3^ in the Caco-2 and 2.01 ± 1.64 cm^3^ in the NIH/3T3 group. The tumors derived from NIH/3T3 cells grew significantly slower compared to Caco-2-derived tumors. Due to cachexia, in the Caco-2 group the absolute organ weights were significantly lower compared to that of the other groups whereas the relative organ weights were mostly higher due to more rapid decrease of the body weight compared to the organ weight (Additional file 2: Figure S2). The absolute and relative organ weight of the mice with tumor-suspicious organs or tissues did not differ from that of other mice. Thus, examination of organ or tissue weights (Fig. 2, method VII) was not useful to detect tumor growth outside spheroid implantation site.

### Final evaluation of the tumorigenicity study

Overall, examining tumorigenicity by combining methods ensures safe testing of human ATMPs in immunodeficient NSG mice and resulted in cost savings. However, the weight measurement of organs was not sensitive enough to detect small tumors. There were no indications of increased prevalence of tumors after implantation of huChon spheroids in the applied NSG mouse model.

## Discussion

The aims of the present study were first to design a strategy to evaluate biodistribution and tumorigenicity of ATMPs without previous cell manipulation, implementing considerations and demands of the regulatory authority, and secondly, to apply this approach in the assessment of safety issues of the human cartilage ATMP co.don chondrosphere®. These non-clinical studies are necessary for central market authorization in the European Union.

To the best of our knowledge, this is the first report on a comprehensive GLP safety and efficacy study on a human ATMP for cartilage repair. Until now, only limited non-clinical data have been published regarding safety of cell-based medicinal products [22]. Our study delivers relevant non-clinical safety data for the investigated TEP. Even more important, it provides an example of a comprehensive and validated testing strategy which is in agreement with EMA and FDA guidelines for non-clinical safety testing of TEPs, sCTMPs and combined ATMPs under GLP.

Xenogeneic graft placement of human ATMPs requires an immunodeficient animal model. The NSG mouse expresses one of the most pronounced immunodeficient phenotypes [23] and represents the most widely used model available for studying human xenografts in mice *in vivo* [24]. Moreover, the induction of tumors after implantation of various cancer cell lines was higher in NSG compared to other immunodeficient mouse strains such as SCID, NOD-SCID or NSB [25,26]. In agreement with the PEI, we favored a heterotopic variant for spheroid implantation because of the murine knee anatomy. Although intra-articular injection of fibroblasts into the knee joint is feasible in mice [27] this invasive procedure may destroy regular structures in the knee joint. In contrast, subcutaneous implantation is easier to perform, is non-invasive, and offers the advantage that several test items may be applied in one mouse [28].

To test biodistribution, xenogeneic cells in mouse organs were quantified by analyzing species-specific, non-coding DNA sequences using qPCR [7]. In the present study, sensitivity using the Plexor^®^ HY system was 5 cells per 50 mg mouse tissue which corresponded to 0.96 cm^2^ back skin. Thus, five cells could be detected safely within 0.66 cm^2^, the area of the 10-mm skin ring. Based on our results for macro- and microscopic evaluation and qPCR, there was no biomolecular evidence of biodistribution of human chondrocytes or huChon spheroids away from the implantation site. Generally, chondrocytes are able to move in 2D and 3D environments *in vitro* with a speed of 1-15 μm/h [29,30]. Although *in-vivo* migration is more complex, based on these data, migration up to 11.2 mm distant from the spheroid and thus into the 20-mm skin ring might be possible within one month. However, this was not evident. The few HLA-ABC positive 10-mm skin rings were most likely caused by partial deformation or fragmentation of the soft spheroid and were judged as false positives. Correspondingly, the data from an assessment report for approval of “Matrix applied characterized autologous cultured chondrocytes” (MACI®, Genzyme/Sanofi) to the EMA revealed no biodistribution from the orthotopic implanted cells into popliteal and ileofemoral lymph nodes in a preclinical horse model (Study GENZ 09–4417, [13]).

A limitation of the prospectively defined biodistribution testing strategy is that only 23/40 huChon spheroids could be recovered unequivocally by IHC. The loss of the test item during processing for IHC was likely due to the soft consistency of the analyzed ATMP.

To overcome this detection bias, an additional random sample of the organs of 7 mice out of the 17 mice which were immunohistochemically not evaluable was analyzed more extensively by qPCR (Additional file 3: Figure S3). In this random sample, no biodistribution was observed. In future non-clinical studies regarding testing of similarly small and soft test items, the processing of the samples for IHC should be carefully adjusted in order to maximize the number of animals evaluable by IHC.

In the tumorigenicity study, the huChon spheroids but no tumors and metastases were detected at the implantation site of all mice after six months. Because the previous biodistribution study yielded no signs of migration from the huChon spheroids one month after implantation, tumor formation outside the implantation site was not anticipated. However, in two mice HLA-ABC-negative benign tumors were detected, indicating that these tumors were of murine origin and occurred spontaneously without relation to the huChon implantation. Data on the incidence of spontaneous tumors are not available for NSG mice but are available for other mouse strains [31]. In 17/32 inbred mouse strains, the liver was one of the organs which was often affected [31]. As an example, in immunodeficient C.B–17;ICR-Prkdcscid (SCID) mice, spontaneous liver tumors were observed in 0–14 % of the animals. Thus, the liver tumor incidence of 2.6 % in our study was within this range. Spontaneous intestinal tumors appeared in 14/32 of the inbred mouse strains [31]. In our study, the incidence of such tumors was 2.6 % and thus comparable to that in the literature [32]. Moreover, the formation of spontaneous hepatomas, observed once in our study, increased during the lifetime of inbred mouse strains and was nearly 100 % in 120-week old animals [33].

In the tumorigenicity study we included two reference cell lines. As a positive reference which would certainly induce tumors after subcutaneous implantation into the neck, we would have preferred chondrocyte-derived malignant cells [34], but the murine OUMS-27 chondrosarcoma [35] proliferated too slowly and did not form spheroids *in vitro* according to the co.don protocol. After expert advice from the PEI, the German responsible authority, we used Caco-2 cells as a positive reference, as they proliferate faster and form spheroids. For the second reference, NIH/3T3 cells planned as negative reference, tumor growth in mice was unexpected when formulating the study plan. These cells did not develop tumors in SCID mice in an internal pilot study. It is likely that NSG mice might be more sensitive for tumor formation. However, the accumulated chromosomal anomalies of the NIH/3T3 cells used in this study were considered to be the predominant cause of NIH/3T3-related tumor growth in our *in-vivo* model. Thus, the NIH/3T3 cell line is not a suitable negative reference control for testing tumorigenicity of ATMPs. In conclusion, cell lines with an abnormal karyotype bear the risk of tumor formation, particularly in immunocompromised animal models. Freshly isolated primary fibroblasts would provide an ideal but not feasible or reproducible cellular source as a negative reference.

## Conclusions

In summary, we developed a strategy to study the safety of human ATMPs under GLP conditions. The analysis of the chondrocyte-based TEP co.don chondrosphere® revealed no risk for biodistribution. The subsequent tumorigenicity study also revealed no indications of increased tumor frequency in the applied NSG mouse model. The step-wise, sequential strategy presented here lowers personnel and material costs of expensive safety testing. This strategy might serve as a good basis for non-clinical safety studies of other solid cell-based ATMPs in a mouse model, including TEPs, sCTMPs and combined ATMPs.

## Additional files

Additional file 1: Figure S1. Pilot batch for the development of the immunohistochemical staining procedure of huChon spheroids within mouse skin. Blocking of endogenous peroxidase (+) showed a significantly reduced background compared to non-blocked sections **(A)** in the area of epidermis/hair follicles as well as in the deeper layer around the implanted spheroid (arrow head). Besides blocking of endogenous peroxidase, the sections were blocked with different concentrations of human serum (HS) before incubation with the primary anti-human antibody **(B)**. 1 % HS was the best. The non-specific background (arrows) increased with HS concentration. Additional file 2: Figure S2. Organ weights of mice in the tumorigenicity study. Absolute organ weights **(A)** of the three groups and relative organ weights **(B)** as a percentage of body weight (BW). Error Bar Chart (mean ± 2 SD) of tumor size by group. Note: ± 2 SD covers approx. 95 % of all data in the sample. Additional file 3: Figure S3. Biodistribution study: Flow chart of the 4-mm skin discs that could not be evaluated by IHC due to technical/product specific reasons. The steps with continuous lines were performed according to the study plan. The dashed lines indicate the organ analysis by qPCR, performed additionally to the study plan on seven random samples (including mouse MB1). This was performed to further increase the level of security. The Roman numerals have been adopted from the biodistribution flow chart (Fig. 1). ^a^A total of 102 organs were isolated from all 17 mice. From these, a sample of 40 organs was analyzed by qPCR (see section of qPCR results). The 40 analyzed organs are calculated as follows: 7 mice × 5 organs (lungs, liver, left and right kidney, spleen) + local lymph nodes of 5 mice. Due to the very small lymph nodes of the NSG mouse [31] they could only be isolated from 5 out of 7 mice.

## Abbreviations

ATMP: Advanced therapy medicinal product
EMA: European medicines agency
GMP: Good manufacturing practice
PEI: Paul-Ehrlich-Institute (Federal Institute for Vaccines and Biomedicines, Langen, Germany)
sCTMP: Somatic cell therapy medicinal product
TEP: Tissue engineered product
