# Supplementary figures and images for: Preclinical good laboratory practice-compliant safety study to evaluate biodistribution and tumorigenicity of a cartilage advanced therapy medicinal product (ATMP)
Source: J Transl Med. 2015 May 20;13:160. doi: 10.1186/s12967-015-0517-x (PMC4445304; doi:10.1186/s12967-015-0517-x)

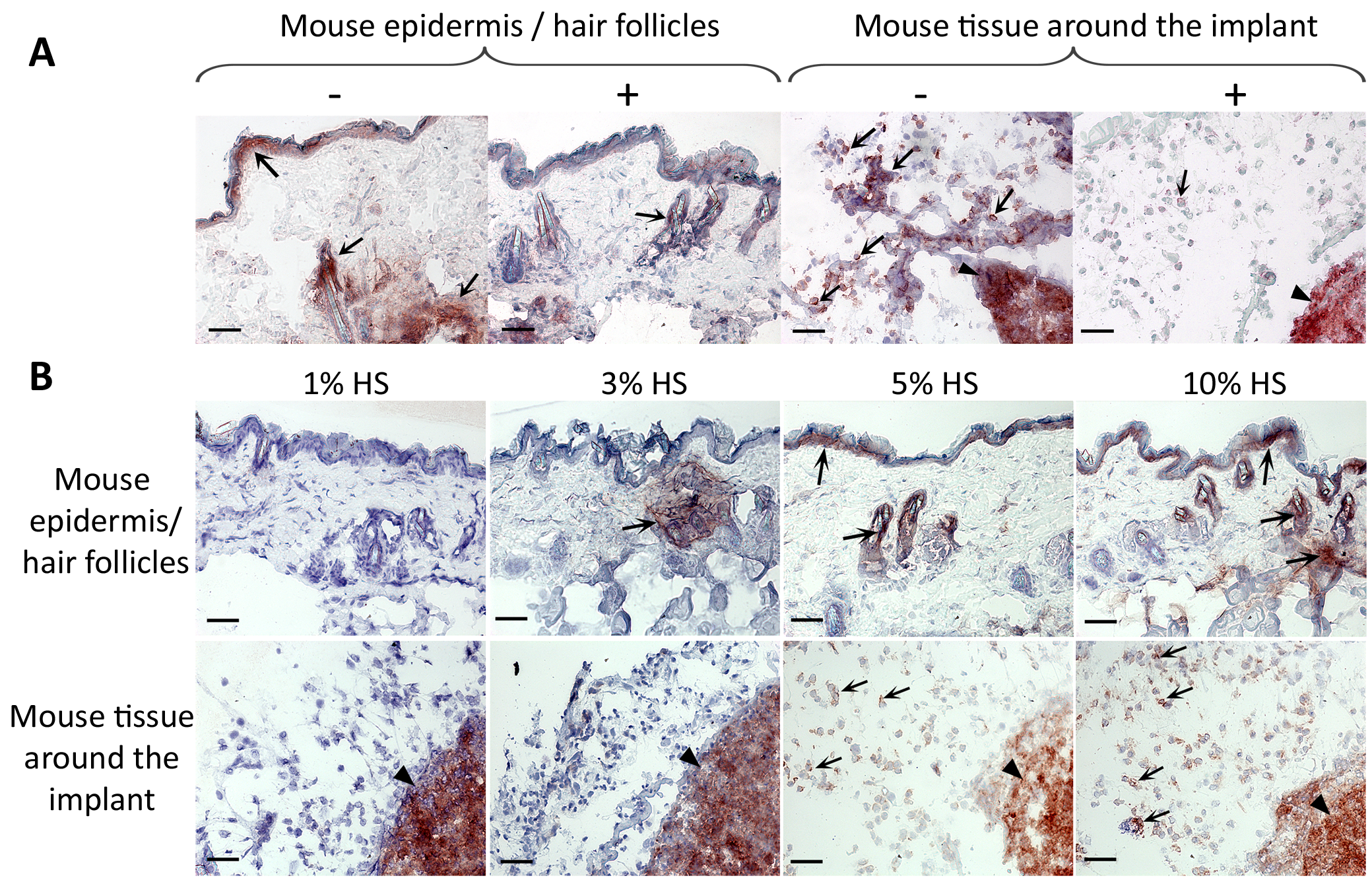

Supplement: Additional file 1: Figure S1. — Pilot batch for the development of the immunohistochemical staining procedure of huChon spheroids within mouse skin. Blocking of endogenous peroxidase (+) showed a significantly reduced background compared to non-blocked sections (A) in the area of epidermis/hair follicles as well as in the deeper layer around the implanted spheroid (arrow head). Besides blocking of endogenous peroxidase, the sections were blocked with different concentrations of human serum (HS) before incubation with the primary anti-human antibody (B). 1 % HS was the best. The non-specific background (arrows) increased with HS concentration. [file 12967_2015_517_MOESM1_ESM.tiff]

**A**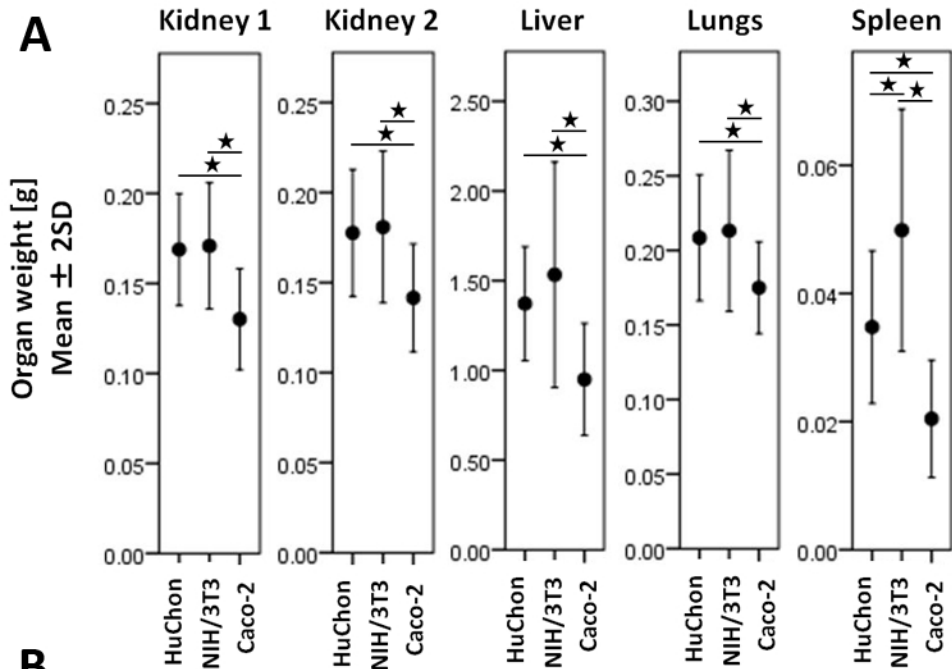**B**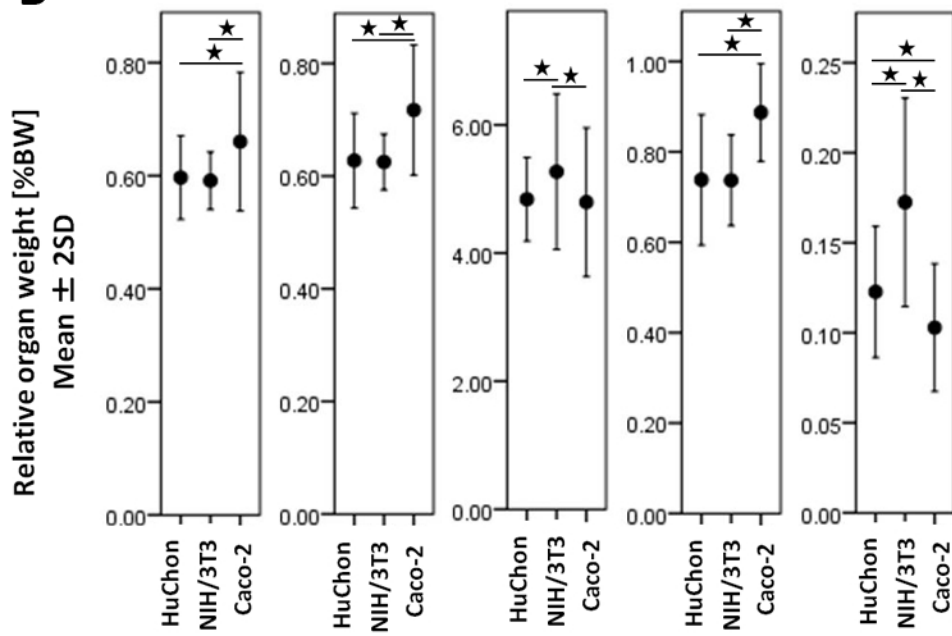

Supplement: Additional file 2: Figure S2. — Organ weights of mice in the tumorigenicity study. Absolute organ weights (A) of the three groups and relative organ weights (B) as a percentage of body weight (BW). Error Bar Chart (mean ± 2 SD) of tumor size by group. Note: ± 2 SD covers approx. 95 % of all data in the sample. [file 12967_2015_517_MOESM2_ESM.pdf]

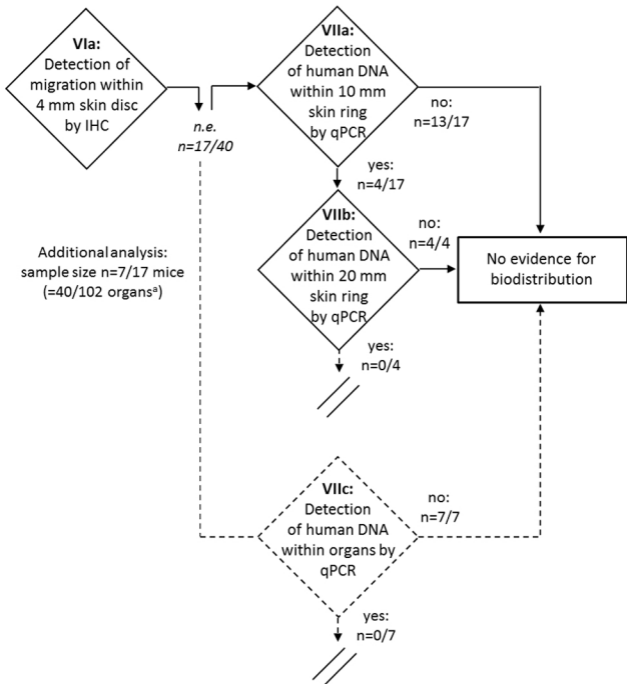

Supplement: Additional file 3: Figure S3. — Biodistribution study: Flow chart of the 4-mm skin discs that could not be evaluated by IHC due to technical/product specific reasons. The steps with continuous lines were performed according to the study plan. The dashed lines indicate the organ analysis by qPCR, performed additionally to the study plan on seven random samples (including mouse MB1). This was performed to further increase the level of security. The Roman numerals have been adopted from the biodistribution flow chart (Fig. 1). aA total of 102 organs were isolated from all 17 mice. From these, a sample of 40 organs was analyzed by qPCR (see section of qPCR results). The 40 analyzed organs are calculated as follows: 7 mice × 5 organs (lungs, liver, left and right kidney, spleen) + local lymph nodes of 5 mice. Due to the very small lymph nodes of the NSG mouse [31] they could only be isolated from 5 out of 7 mice. [file 12967_2015_517_MOESM3_ESM.pdf]
